# Identification of a predominant genotype of *Mycobacterium tuberculosis* in Brazilian indigenous population

**DOI:** 10.1038/s41598-020-79621-3

**Published:** 2021-01-13

**Authors:** S. A. Hadi, I. V. Kolte, E. P. Brenner, E. A. T. Cunha, V. Simonsen, L. Ferrazoli, D. A. M. Villela, R. S. Santos, J. Ravi, S. Sreevatsan, P. C. Basta

**Affiliations:** 1 College of Veterinary Medicine, Michigan State University, 784 Wilson Drive, East Lansing, MI 48824 USA; 2 Escola Nacional de Saúde Pública, Oswaldo Cruz Foundation, Rua Leopoldo Bulhões, 1480, Rio de Janeiro, RJ 21041-210 Brasil; 3 Laboratório Central de Saúde Pública de Mato Grosso do Sul, Campo Grande, Brasil; 4 Adolfo Lutz Institute, São Paulo, Brasil; 5 Programa de Computação Científica, Fundação Oswaldo Cruz, Rio de Janeiro, Brasil

**Keywords:** Bacteriology, Genomics, Comparative genomics, Genome evolution, Phylogenomics, Evolution, Genetics, Microbiology, Medical research, Respiratory tract diseases, Infectious diseases, Bacterial infection

## Abstract

After nearly a century of vaccination and six decades of drug therapy, tuberculosis (TB) kills more people annually than any other infectious disease. Substantial challenges to disease eradication remain among vulnerable and underserved populations. The Guarani-Kaiowá people are an indigenous population in Paraguay and the Brazilian state of *Mato Grosso do Sul*. This community, marginalized in Brazilian society, experiences severe poverty. Like other South American indigenous populations, their TB prevalence is high, but the disease has remained largely unstudied in their communities. Herein, *Mycobacterium tuberculosis* isolates from local clinics were whole genome sequenced, and a population genetic framework was generated. Phylogenetics show *M. tuberculosis* isolates in the Guarani-Kaiowá people cluster away from selected reference strains, suggesting divergence. Most cluster in a single group, further characterized as *M. tuberculosis* sublineage 4.3.3. Closer analysis of SNPs showed numerous variants across the genome, including in drug resistance-associated genes, and with many unique changes fixed in each group. We report that local *M. tuberculosis* strains have acquired unique polymorphisms in the Guarani-Kaiowá people, and drug resistance characterization is urgently needed to inform public health to ensure proper care and avoid further evolution and spread of drug-resistant TB.

## Introduction

The WHO EndTB strategy focuses on the reduction of incidence and mortality rates towards the elimination of tuberculosis (TB) by 2035, yet the disease remains a substantial public health threat worldwide. Ethnic minorities who face precarious living conditions and lack access to healthcare are at greater risk. TB eradication in aboriginal (indigenous) groups is further complicated by sparse data collection from these communities. The existing evidence indicates that incidence of active TB and prevalence of latent TB infection (LTBI) are significantly higher in indigenous groups compared to non-indigenous populations globally^1^. This is a result of socioeconomic inequalities, including limited access to education, lack of employment opportunities and marginalization^2^. To achieve TB eradication in these communities, high-resolution surveillance tools are needed. The use of whole-genome sequencing (WGS) in TB research provides the highest possible resolution to study disease transmission. WGS can diagnose infections directly from primary clinical samples, provide results faster, predict antimicrobial agent resistance-associated genotypes, and has applications in outbreak investigations to better define transmission clusters^3–8^.

Despite significant advances in TB control in recent decades, Brazil remains a high-burden country with an estimated 95,000 new cases of TB diagnosed in 2018^9^. Indigenous groups in Brazil suffer from incidence rates three times than that of the general population^10^. Studies have suggested nearly 50% LTBI prevalence rates in some indigenous territories, along with evidence of recent and ongoing transmission^11,12^. The Guarani-Kaiowá people in the Brazilian state of *Mato Grosso do Sul* (MS) are Brazil’s second largest indigenous group at around 44,000 individuals. For the last decade, MS has borne the country’s highest rate of TB among its indigenous population. In 2013, a study of the indigenous people in MS found annual incidence rates of diagnosed active TB to be six times the state average^13^. The southern part of the state, Amambai district, stood out with an average annual TB incidence rate of around 400/100,000 in the population of Guarani-Kaiowá ethnicity^13^.

The aim of this study was to characterize the genomic patterns of diversification of drug resistance, and explore whether hyperendemic, locally circulating *Mycobacterium tuberculosis* in the indigenous population of the Guarani-Kaiowá may be a product of regular introductions from outside the community, or unchecked transmission within.

## Results

### Characterization of the patient population

Selected samples reflected the characteristics of the overall TB patient population in the study area^14,15^ (Supplemental 1—Study Characteristics). Diagnosed patients were 62% male (71% of patients from whom we obtained WGS data). TB patients were relatively young, with a median age of 32.5 in the study area (33 years for our subset used WGS analysis). Nearly 50% of patients sought healthcare within a week of symptom onset, but around 20% (in total and WGS subset cases) reported waiting five weeks or longer to seek healthcare. 9/68 patients had been treated for TB one or more times in the past.

### Whole-genome sequencing

After genome processing, the 414 lineage-determining single-nucleotide polymorphisms (SNPs) published in Coll et al*.* were utilized^16^. Four main groups emerged from the analysis (Supplemental 2—SNP Barcoding for Lineage identification). The dominant sublineage (n = 54/66, Supplemental 2, col. D:BE, blue) was 4.3.3. The 4.3.3-determining *Rv1248c* C2526G was always paired with an adjacent G2525T mutation (S842Y). This SNP, among others, may suggest divergence of sublineage of 4.3.3 in Brazil that warrants further exploration. In total, 97% of all M. tuberculosis analyzed in this study was identified as sublineage 4.3. When analyzing 9 Brazilian isolates from another study as local references, only 2 out of 7 that were classified as sublineage 4.3 (local references 3 and 5) shared some of the SNPs fixed in our dataset. Separately, 61 SNPs found at greater than 80% prevalence in our isolates were completely absent in all global and local references, including in local references 3 and 5. Remaining isolates belonged to sub-lineages 4.3.4.2 (n = 9/66, Supplemental 2, col. BG:BO, green), 4.4.1.1 (n = 2/66, col. BP:BQ, red), and ambiguous S58 (col. BF, yellow) in either 4.3.2 or 4.3.3, carrying an unusual mixed SNP genotype from multiple lineages (although *Rv1248c* G2525T/C2526G was absent).

**Figure 1:**
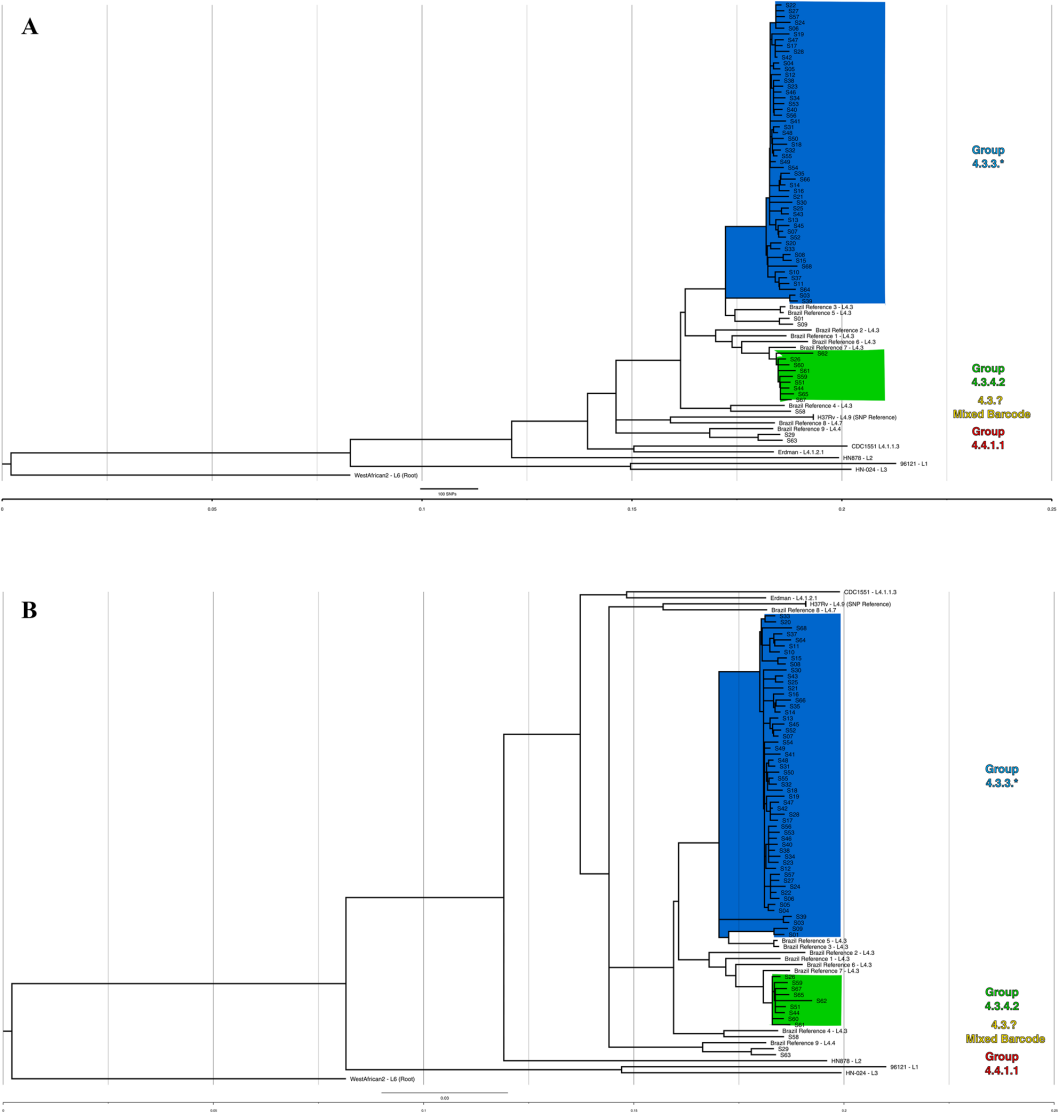

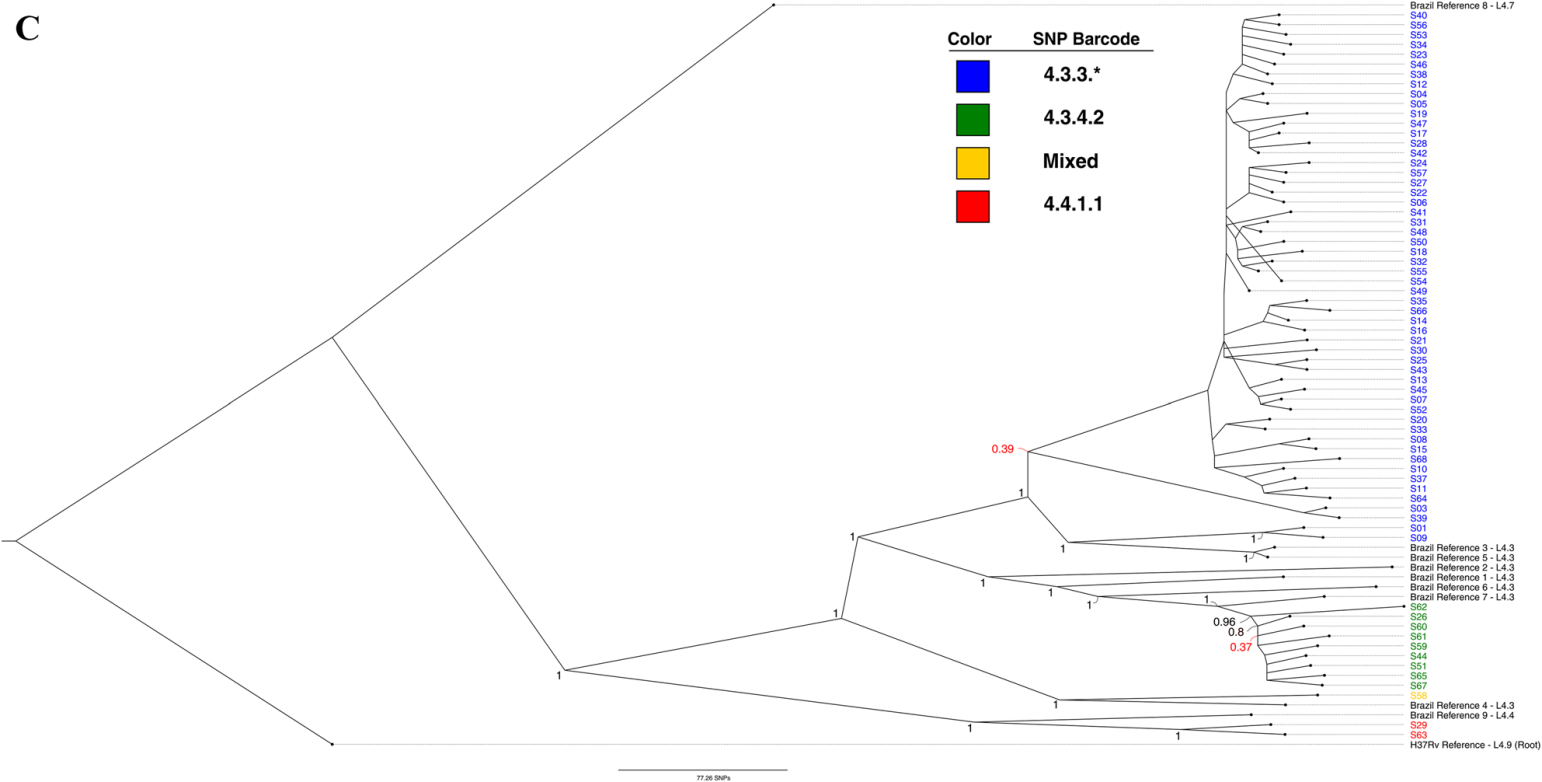
(**A**) Maximum likelihood (ML) tree showing SNP-based phylogenetic analysis of core SNPs extracted by snippy. Analysis by MEGA-X (default parameters, HKY model) with 500 bootstrap replicates. Tree with highest log likelihood is shown (− 53,357.07). Tree was visualized in FigTree, and labeling generated in Inkscape software. The tree is rooted to *M. tuberculosis* GM041182 (West African 2). The tree is drawn to scale, with branch lengths measured in average substitutions per site (7420 sites total). Bootstrap values are robust across the entire tree and are available in Supplemental 4. Pairwise distances are available in Supplemental 3. Coloring represents groups identified by Coll et al*.*’s TB barcoding strategy, that groups the isolates based on the presence or absence of lineage-determining SNPs (Supplemental 2). The tree cleanly groups similarly barcoded TB isolates and suggests divergence of sampled isolates even from recent local reference isolates. (**B**) Bayesian consensus phylogram showing SNP-based phylogenetic analysis of 7420 core SNPs extracted by snippy. Analysis performed by MrBayes (GTR model, nst = mixed, mcmc = 500,000). Tree was visualized in FigTree, and labeling generated in Inkscape software. The tree is rooted to *M. tuberculosis* GM041182 (West African 2). All tree statistics and raw files are available in Supplemental 5. Coloring represents groups identified by Coll et al*.*’s TB barcoding strategy available in Supplemental B. The tree demonstrates nearly identical grouping of all isolates as the maximum likelihood tree in (**A**) and lends even more support for barcoding-based grouping. (**C**) Maximum likelihood (ML) tree showing SNP-based phylogenetic analysis of core SNPs extracted by snippy without global reference lineages. Analysis by MEGA-X (default parameters, HKY model) with 1000 bootstrap replicates. Tree with highest log likelihood is shown (− 30,203.04). Tree was visualized in FigTree, and labeling generated in Inkscape software. The tree is rooted to *M. tuberculosis* H37Rv (Lineage 4.9). The tree is drawn to scale, with branch lengths measured in average substitutions per site (3863 sites total). Bootstrap values are labeled at branch points if greater than 70%, and any points where support falls under 70% is labeled in red and further values are not provided for the clade. Coloring represents groups identified by Coll et al*.*’s TB barcoding strategy available in Supplemental 2. Without global reference lineages, barcoded isolates still group reliably. S09/S01 and S03/S39 (blue clade, bottom four isolates) stand out by branch lengths as divergent from rest of blue clade.

A maximum likelihood (ML) phylogenetic tree was generated in MEGA-X to identify genotype clustering patterns. The tree with the highest log likelihood is shown in Fig. 1A. Branch lengths are drawn to scale and represent the number of substitutions. All isolates fell well-separated from global *M. tuberculosis* references [closest = H37Rv, > 600SNPs avg. pairwise distance (Supplemental 3—Pairwise distance))]. Bootstrap values were well above recommended cutoffs for statistical support^17^. An additional maximum likelihood analysis’ tree files, including a tree with bootstraps labeled, are provided in Supplemental 4. The blue clade representing 4.3.3 contains most isolates (n = 54/66, ~ 82%), though four of these isolates (S01/S09, and S03/S39) stand on relatively distant, robust branches and appear distinct. These isolates fell at least ~ 180 SNPs away from closest local reference (local reference 5 to S01). The green clade, 4.3.4.2, consisted of 9 isolates, grouped next to four local references (local references 1, 2, 6, and 7), but still showed a minimum SNP distance of ~ 75 SNPs. The red clade (S63/S29) grouped with local reference 9 at ~ 245 SNPs distance. Finally, S58 (yellow clade) grouped ~ 200 SNPs away from closest local reference 4.

Next, a Bayesian phylogenetic consensus tree was generated in MrBayes and is shown in Fig. 1B. The tree closely replicates the reconstruction by the ML tree, and all results from the previous tree apply. Support for the run and Bayesian tree were excellent (Supplemental 5). Finally, an additional ML tree built against H37Rv with only local strains is shown in Fig. 1C, providing an at-a-glance visualization of how barcoding aligned with phylogenetic grouping.

Both the barcoding strategy and phylogenetic trees using different methodologies produced nearly identical findings. Over 80% of isolates grouped into a dominant clade typed as 4.3.3, and some distance was observed even between recent Brazilian strains from another study and the isolates circulating in the studied population.

### Single-nucleotide polymorphism analysis and drug resistance-associated genes

Of 68 patients, 9 had been treated for tuberculosis at least once before. One of these isolates failed QC (S02), but of the remaining 8, 6 were infected by 4.3.3, and 2 by 4.3.4.2. To investigate potential drug resistance in this community, an analysis of 13 drug resistance (DR) associated genes was performed, revealing 24 single nucleotide polymorphisms (SNPs). Of these, 79.2% (19/24) led to missense mutations. Among these missense mutations, 57.9% (11/19) were unique to the Guarani-Kaiowá isolates when compared against the 9 Brazilian isolates from Brynildsrud et al.^18^. Details of each SNP for every drug resistance-associated gene analyzed for all isolates can be seen in Supplemental 6 (SNP Summary: Sheet 2), which were extracted from the core SNPs (Supplemental 6—SNP Summary: Sheet 1).

Pyrazinamide resistance is associated with *pncA*^19,20^*.* All isolates in our dataset appeared genotypically sensitive to pyrazinamide. Additionally, streptomycin resistance-associated genes *rpsL* and *rrs* were found to be wild-type.

**Figure 2:**
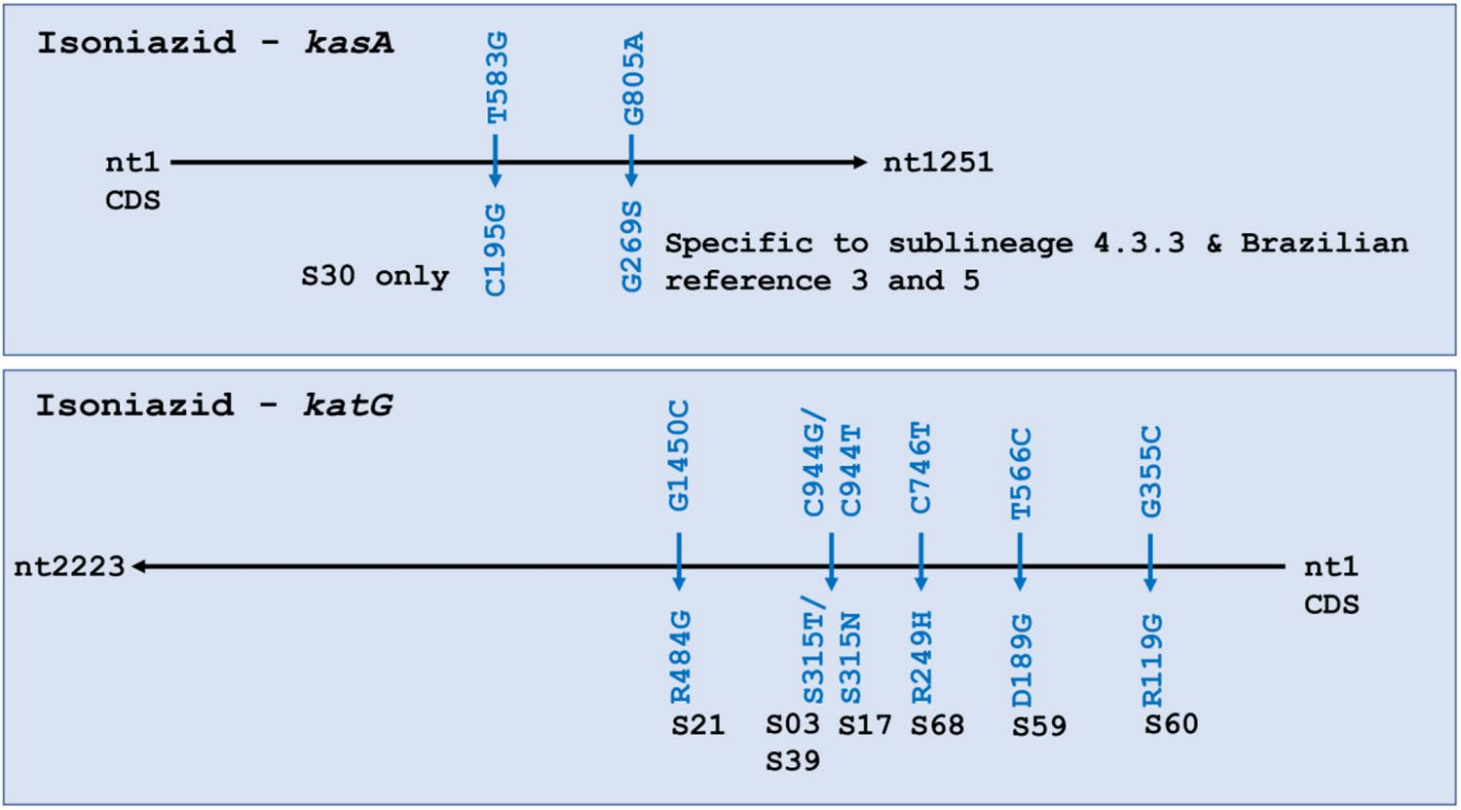
Isoniazid resistance-associated genes *katG* and *kasA* had multiple single nucleotide polymorphisms, whereas *ahpC* and *inhA* were wildtype. Missense mutations are colored blue.

Isoniazid resistance is associated with *inhA*, *ahpC*, *kasA,* and *katG*^21,22^. All four genes were analyzed for variants, including 100 bp upstream of the start codon for *inhA*. No SNPs were seen in *inhA* or *ahpC*. Two SNPs were identified in *kasA* and six in *katG*. These eight SNPs all led to missense mutations (Fig. 2). In *kasA*, G805A was present in all 54 isolates in 4.3.3. This SNP was shared only by local references 3 and 5. In *KatG*, two mutations were found in three isolates at nucleotide C944, one leading to S315T (S03, S39) and the other to S315N (S17). This SNP was also found to be commonly circulating in all local reference strains except in local reference 1. Only two samples in our dataset were known to be phenotypically resistant to isoniazid—S03 and S17—and both had SNPs in *kasA* and *katG.*

**Figure 3:**
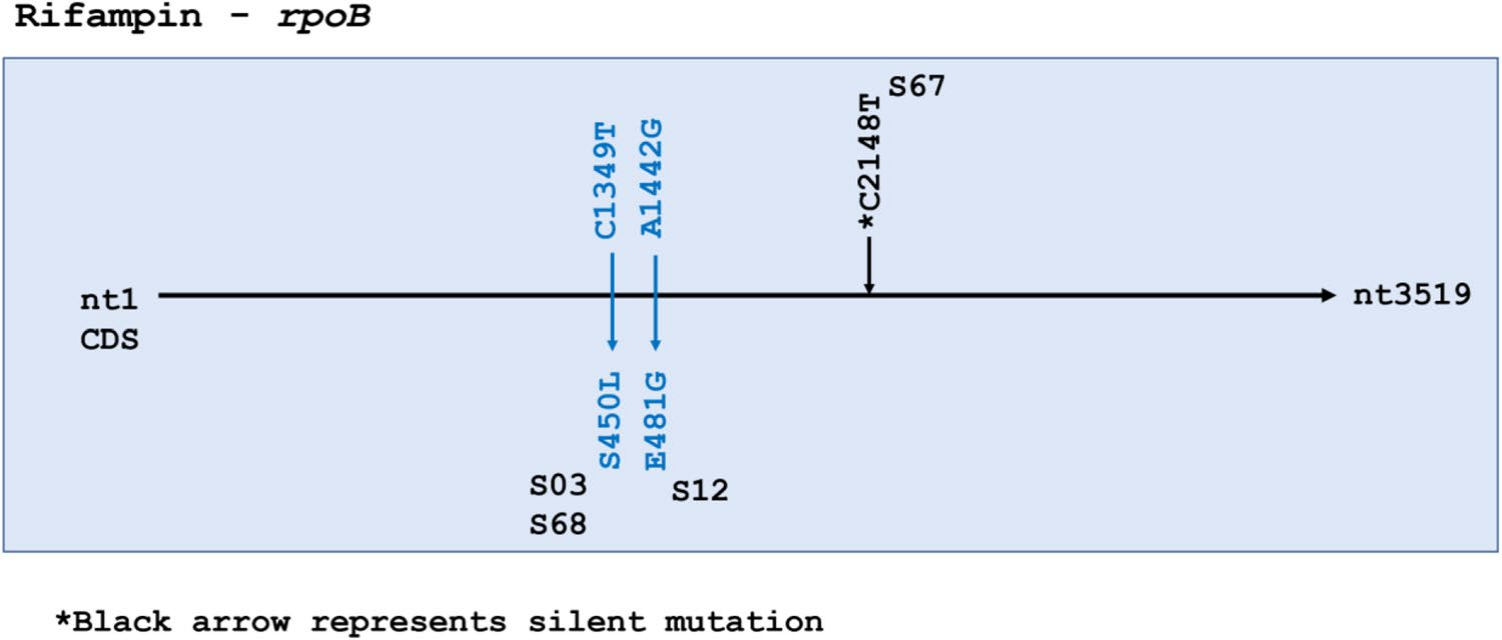
Rifampin resistance-associated gene *rpoB* had only three single nucleotide polymorphisms (SNPs). Missense mutations are colored blue. S67 had a silent mutation represented by the *black arrow.

The 80 bp region in *rpoB*—the rifampicin resistance-determining region (RRDR)—did not carry any mutations However, two missense mutations were seen outside the RRDR in three samples—S03, S68 and S12 (Fig. 3). S03 was the only sample that was phenotypically rifampin resistant, confirming S03 as multidrug resistant both phenotypically and genotypically due to presence of mutations in *kasA*, *katG*, and *rpoB*, although the *rpoB* SNP fell outside the RRDR. No mutations were observed in *rpoA* in any of the isolates*,* while S68 showed a missense mutation C3119G (P1040R) in *rpoC*. It is of note that S50 was phenotypically resistant to rifampin, but no SNPs were identified in *rpoB*.

**Figure 4:**
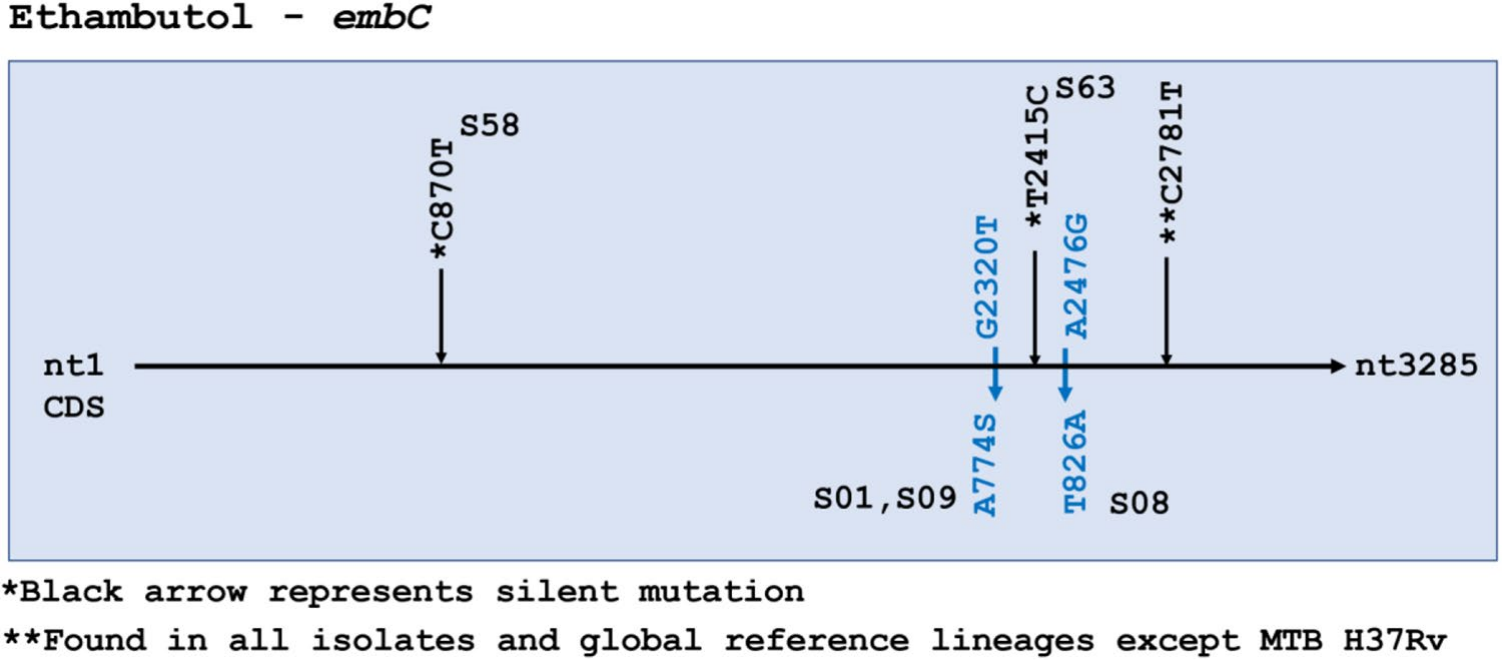
Ethambutol resistance-associated gene *embC* had 2 missense single nucleotide polymorphisms (SNPs) and one silent SNP (**C27181T) found in all 66 isolates in the data set as well all reference strains except H37Rv and is not considered particularly informative. All samples were wildtype for *embA.* Missense mutations are colored blue. Silent mutations are represented in *black color.

Ethambutol resistance-associated mutations occur in three genes identified as *embABC*. No SNPs were detected in *embA*. One silent mutation in *embB*, and 3 silent plus 2 missense mutations in *embC* were identified (Fig. 4). *embC* missense mutations were seen in S01, S09 and S08, but drug susceptibility data was not available for any of these isolates. The silent mutation at C2781T in *embC* was found in all isolates and references except H37Rv.

**Figure 5:**
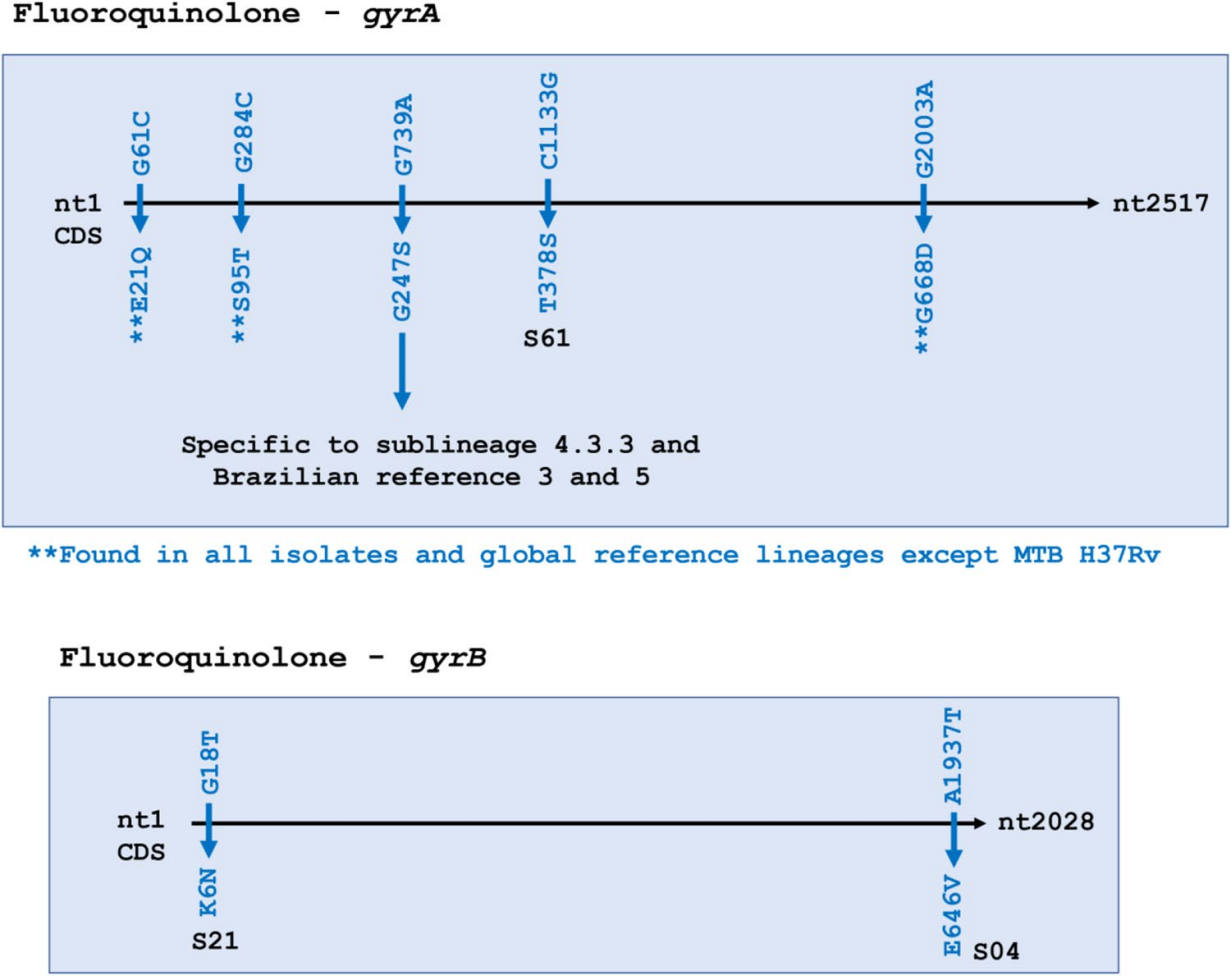
Fluoroquinolone resistance-associated genes *gyrA* and *gyrB*, had a few yet significant single nucleotide polymorphisms (SNPs). All SNPs were missense (blue). Three SNPs (**) in *gyrA* were fixed in the entire data set and all references except H37Rv, whereas one SNP in *gyrA* was found in 54/66 isolates. The former are not considered particularly informative, while the latter does appear to be. Two SNPs were observed in *gyr*B.

For the second line anti-tuberculosis drug fluoroquinolone, two genes—*gyrA* and *gyrB*—were analyzed. All SNPs identified were missense—5 in *gyrA* and 2 in *gyrB*. Out of the 5 SNPs in *gyrA*, G739A (G247S) was fixed in all 54 isolates that fell in sublineage 4.3.3. Three SNPs were common to all 66 isolates as well as our global references except for H37Rv: *gyrA* E21Q, S95T, and G668D. These 3 SNPs were present in almost all Brazilian reference strains, with the exception of S95T & G668D that were absent in local reference 8. The 2 missense SNPs in *gyrB* were found only in two isolates and were absent in Brazilian references (Fig. 5).

## Discussion

Although TB is hyperendemic among indigenous populations like the Guarani-Kaiowá in Brazil, there has been a lack of investigation into TB transmission in these settings, a red flag for public health policy. This is, to our knowledge, the first study applying WGS to TB samples from an indigenous population in a high-burden, low-and-middle-income country. Our phylogenomic study indicates that, although cases were diagnosed across six different territories over a study period of six years, a majority of the sequenced cases are caused by *M. tuberculosis* clustered into sublineage 4.3.3 (Fig. 1, blue clades). The three barcoded sublineages observed showed divergence from global references (~ 600 + SNP average distance), necessitating the inclusion of other recently published Brazilian isolates for comparison (Supplemental 7—Isolates & References: Sheet 3). These nine additional isolates (referred to as “local references”) and an analysis methodology were graciously shared by Drs. Brynildsrud and Eldholm, and this allowed a more refined comparison between Brazilian TB strains^18^. Isolates remain divergent by phylogeny and SNP analysis even when including these local references from 2009 to 2012. While local reference strains reliably cluster with our isolates, the average SNP distance within 4.3.3 isolates was ~ 72 SNPs (SD ± 45), and the distance from two closest 4.3 local references was ~ 210 SNPs (SD ± 10). The strains observed in this study are Latin American-Mediterranean (LAM) type TB, which is itself not rare in the region. However, the unusually high prevalence of *M. tuberculosis* sublineage 4.3 (64/66, ~ 97%) is noteworthy. It is far greater than any LAM incidence observed in any prior report the authors could identify in South America or globally, with frequencies typically up to 50%^23–26^ and peaking in South America at only ~ 65% in one study^24^. We speculate that the robust grouping of isolates distant from global references, the divergence from local references, and the prevalence of a single type of TB (LAM) might represent that disease in this community is not the result of modern, recurring introductions. Rather, mutations absent in global lineages yet fixed in this study potentially suggest earlier, historical introductions of *M. tuberculosis* and a subsequent circulation and diversification of local sublineage 4.3 (LAM) within the Guarani-Kaiowá population. This hypothesis would be expected for an isolated community, but the Guarani-Kaiowá cannot live off their overcrowded territories and are therefore not living in isolation. Our findings then would rather support the extreme level of marginalization suffered by this indigenous group—to the point where the fingerprints of interaction appear largely undetected by high-resolution genomic data.

In both phylogenetic trees, we observed universal agreement for all barcoded groups. Isolates grouped into the blue clades (Fig. 1A,B) are dominant and responsible for most sampled infections. Both trees and SNP-based classifications support additional uncommon subtypes circulating in this population, like the distant sublineage 4.4.1.1 isolates (red clade) of S29/S63, or the unusual genotype observed in S58 (yellow clade). We find 111 SNPs that are absent in global references but common at 80% + prevalence in local references and isolates, an additional 63 SNPs present only in 4.3.3 and the closest related local references 3 and 5, and a further 61 SNPs found at 80% + prevalence solely in our 4.3.3 isolates alone (Supplemental 6—SNP Summary: Sheet 1). Isolates show variability in drug resistance-associated genes, a possible portent of developing resistance. Two variants—*kasA* G805A and *gyrA* G739A—have become dominant and fixed in most sampled infections among the Guarani-Kaiowá population. These fixed variants were specifically identified in isolates of sublineage 4.3.3. Additionally, 4 SNPs were identified in all 66 isolates in the fluoroquinolone (*gyrA*) and ethambutol (*embC*) resistance-associated genes. Without careful genome assembly and associated error-checking of the local reference paired-end reads we used, as well as still more local references to compare against, we cannot say for certain whether all these SNPs are truly unique, nor can we speculate what effects they may have, but they do continue to support diversification within the studied population.

Taken together, this is alarming for the Guarani-Kaiowá people from a public health policy perspective, as patients are currently treated as drug-sensitive unless treatment fails, due to insufficient provision of susceptibility kits to the laboratory environment systems (LACENs). In addition to needless suffering, this may have allowed drug-resistant *M. tuberculosis* to arise unchecked in Guarani-Kaiowá communities. Drug susceptibility testing should be provided by the Brazilian Ministry of Health as part of the national TB control program, as it is urgently needed in these vulnerable populations; both to lessen the existing high disease burden as well as to prevent the emergence of MDR-TB that can cause further harm and confound control efforts.

A recent study of *M. tuberculosis* isolates from the Inuit population in Greenland detected several micro-epidemics that could be confirmed with epidemiological data and noted remarkable geographical confinement of lineages within individual villages^27^. Similar observations have been made among Inuit populations in Canada^28^. The WGS studies among the Inuit have described a pattern of a limited number of endemic lineages circulating since their introduction by white colonizers in the early twentieth century. The occasional outbreaks or micro-epidemics among the Inuit were not driven by an adaptation of *M. tuberculosis* but rather a change in circumstances in the communities^28^ or down-scaling of TB programs following a period of low incidence^27^. A study from New Zealand of *M. tuberculosis* isolates from the Maori population identified L4.4.1.1 sublineage clade of European origin (a sublineage also detected in the current study, albeit at low incidence). Molecular dating showed that this clade was introduced at the time of arrival of European traders. The clade is also found in *M. tuberculosis* isolates from indigenous populations in Canada^29^. While WGS studies of *M. tuberculosis* complex have not yet been carried out among indigenous populations in other regions, high levels of Mycobacterial interspersed repeat units—variable number tandem repeat genotype similarity have also been reported from the Warao in Venezuela^30^. A restriction fragment length polymorphism (RFLP) study from MS using data from 1991 to 2001 showed high genotype similarity^12^, while a later RFLP study from the same area using data from 2009 to 2015 revealed a pattern of more fragmented clustering^31^. This was attributed to a more aggressive policy of LTBI treatment of contacts, thus limiting the reactivation of previously dominant endemic strains^31^.

Our study analyzed genotypic drug resistance patterns in *M. tuberculosis* strains circulating in this small indigenous community. A high prevalence of multiple frontline anti-TB drug-specific mutations identified in this study suggests an urgent need for a change in public policy tailored to this population.

Some isoniazid resistance-associated genes, such as *inhA* or *ahpC,* showed no changes*,* while *katG* and *kasA* had multiple mutations. KatG normally activates the isoniazid prodrug (INH), while KasA contributes to mycolic acid synthesis that isoniazid impairs. Changes to *katG* Ser315 are strongly associated with isoniazid resistance^32^. Globally, a single change in *katG* Ser315 is responsible for 64.2% of all phenotypic resistance to isoniazid, the majority of which (95.3%) cause S315T and a few (3.6%) are associated with S315N^21^. These substitutions were also observed in a study in China, where single *katG* mutations occurred in majority of the cases, most of which had S315T and two bore S315N^33^. In our analysis, S315T was observed in two isolates S03 and S39, while S315N was identified in S17. Though we lack phenotypic data on S39, both S03 and S17 were phenotypically resistant to isoniazid. Jagielski et al*.* reported on SNPs in *kasA* and found that G269S was present in 22% of MDR clinical cases, and in 10% of isoniazid mono-resistant^34^. Older studies have also reported this^32,35–37^. In our work, 81.8% of isolates (54/66) showed G269S (G805A), suggesting a much higher prevalence than found elsewhere. Unfortunately, highly limited phenotypic data (n = 2/54) stymies further interrogation beyond S03 and S17, which were confirmed isoniazid resistant. These findings merit in-depth study of the population, as literature suggests some of the observed and commonplace SNPs are associated with drug resistance and prompting concern about drug-resistant tuberculosis becoming ubiquitous in the Guarani-Kaiowá people.

In *rpoB*, drug resistance-associated mutations are mostly confined to the RRDR (codon 507 to 533), coding for the core of the beta-subunit of RNA polymerase. Almost 95% of all resistance-associated mutations exist in this hotspot. In our study, all 3 SNPs were found to be outside the RRDR. Of 66 isolates, S03 was the only one known to be multidrug-resistant, yet both S68 and S03 had the same amino acid substitution S450L that is commonly associated with resistance to rifampicin^38,39^ This mutation falls inside the polymerase’s highly conserved catalytic pocket (codon 443–451)^40^, suggesting that S68 might also have been multi drug-resistant like S03. Furthermore, S450L has been reported to accumulate significantly more compensatory mutations than other rifampicin resistance-associated SNPs^41^. This was observed in our study uniquely in S68, with a missense mutation in *rpoC* (P1040R) lending further support to possible rifampin-resistance in S68. We lack the phenotypic data to confirm our hypothesis, again calling for intensive drug testing in the community before initiation of treatment^42–44^.

Fluoroquinolone**,** the second-line TB drug, targets a DNA gyrase encoded by *gyrA* and *gyrB*. Like rifampin’s *rpoB* hotspot, drug resistance to fluoroquinolone is also associated with specific mutations in the quinolone resistance-determining region (QRDR) of *gyr*A & *gyrB*^45^*.* In *gyrA*, T378S was a substitution found to be unique to our study (Fig. 5). Another substitution, G247S, was found 100% of all our 4.3.3 isolates and was not seen in any other isolates or global references. Only the two Brazilian reference strains (reference 3 and 5) that grouped on the periphery of our 4.3.3 isolates showed this SNP, suggesting these references may be tightly related to our predominant sublineage. This mutation falls outside the QRDR and has not been associated with fluoroquinolone resistance^46^. Whether it led to drug resistance in patients is unknown, but it is important to note that while indigenous patients are not treated with fluoroquinolones for TB in Brazil, drugs in this major group are regularly administered for other ailments such as pneumonia, foodborne diseases, and urinary tract infections caused by a wide variety of bacteria such as methicillin-resistant *Staphylococcus aureus*^47^, *Enterobacteriaceae*^48^, *Pseudomonas aeruginosa*^49^*,* and *Salmonella*^50^*.* It is possible that the use of fluoroquinolones for other conditions in parallel with endemic TB has led to the development of unique circulating TB strains. Fixing of SNPs in the entire data set and the establishment of unique SNPs beyond what are observed in existing sub-lineages support diversification in the indigenous population.

Multiple mutations were identified in nearly all drug resistance-associated genes that were analyzed, with many in isoniazid, ethambutol, and fluoroquinolone drug resistance-associated genes. A 2000–2006 study analyzing 783 TB isolates (82.4% new cases and 17.6% previously treated patients) from the general population in *Mato Grosso do Sul* found evidence of drug resistance in 18.3% (all combinations) of cases. Looking at single-drug primary resistance, streptomycin showed the highest level of resistance (3.4%), followed by isoniazid (2.9%), ethambutol (1.7%), and rifampin where no resistance was detected. The resistance to streptomycin was believed to be due to endogenous reactivation of TB strains from before the 1980s, as streptomycin was not given to new cases. Acquired resistance was found to isoniazid (7.2%), streptomycin (3.6%), rifampin (2.2%), and ethambutol (1.4%). Although levels of primary resistance were lower than observed in contemporary data from other regions, the levels of acquired resistance were already high in 2000–2006^51^. As the relative risk of TB is six times higher for the indigenous versus the non-indigenous population in MS^10^ we could speculate that a significant proportion of the samples from the 2000–2006 study could have been from indigenous patients.

Though our study lacked lab-confirmed drug resistance data for all samples, ample literature exists that support that the genotypic mutations seen in our work has the potential for resistance to the standard treatment protocol. We recommend that regular surveillance and drug-susceptibility information be made compulsory before the initiation of treatment.

In summary, these data provide the first detailed genomic view of hyperendemic TB circulating within the Guarani-Kaiowá people. The vast majority of *M. tuberculosis* isolates fell into a dominant clade of the Latin American sub-lineage 4.3.3 by Coll et al*.*’s SNP barcoding strategy^16^. This grouping was fully supported by two phylogenetic methods using a core SNP set of 7420 total positions taken from our 66 isolates, 7 global references, and 9 local references. Analysis of SNPs both for drug resistance-associated genes—specifically *gyrA* (fluoroquinolone) and *kasA* (isoniazid)—and for lineage-determination revealed diversification, with unique SNPs fixed in the population. Among 4.3.3-classified isolates, there was the universal presence of a two nucleotide *Rv1248c* G2525T/C2526G change. Unlike 4.3.3 sub-lineage determining *Rv1248c* C2526G*,* these two changes lead to an amino acid substitution (S842Y). When looking at references, this substitution was only observed in 2/7 of Brazilian 4.3 references. This and the presence of other unique SNPs fixed in the population (*gyrA* and *kasA*) leads us to speculate that most TB in the Guarani- Kaiowá people is the result of a historical introduction of the commonly circulating 4.3.3 lineage, with subsequent diversification in the community. The incidence of sublineage 4.3 is far higher than observed anywhere else in South America, suggesting it is hyperendemic in the population. Molecular and phenotypic characterization of this microevolution is important to discern whether drug resistance has become fixed in this community. The widespread SNPs observed in this study suggest that a more targeted public health strategy should be implemented in the region, where TB cases are not screened for drug resistance unless all prior treatments have failed.

## Methods

### Study area, population, and design

The population of the Brazilian state of MS is around 2.7 million, including approximately 74,000 indigenous people from nine different groups, of which the Guarani-Kaiowá and the Terena ethnicities represent the majority. Despite international condemnation of human rights violations against the Guarani-Kaiowá^52^, the Guarani-Kaiowá remain confined to overcrowded reservations^53^. Most income for the Guarani-Kaiowá in southern MS comes from seasonal work on plantations, where overcrowded workers’ barracks have been identified as TB transmission hotspots^54^. Poor living conditions of the Guarani-Kaiowá, with high rates of violence^53^, low education and income levels^55^, and food insecurity^56^ favor transmission of TB.

Control of TB in the indigenous populations of Brazil is the responsibility of the Special Secretariat for Indigenous Health of the Ministry of Health (Portuguese: SESAI). SESAI is composed of 34 Special Indigenous Health Districts which organize healthcare centers located in areas with significant indigenous populations. Each center manages smaller healthcare stations inside each indigenous territory as primary points of care. We examined TB cases from the *Amambai* and *Caarapó* healthcare centers during the study period of 2011 until 2016 (Supplemental 1—Study characteristics).

TB diagnosis in the study area was carried out at local healthcare centers. Ziehl–Neelsen bacilloscopy was performed until 2014 when GeneXpert took its place. Ministry of Health’s TB protocol^57^, positive bacilloscopy/GeneXpert results should be confirmed by the culture at the General Laboratory of MS; this confirmation is not always possible due to logistical challenges in getting samples to the laboratory^58^ on top of well-documented difficulties with successfully culturing *M. tuberculosis* ^59^.

Per the Ministry of Health^57^, the standard treatment regimen for TB during the study period was two months of rifampin, isoniazid, pyrazinamide, and ethambutol, followed by four months rifampin and isoniazid. Streptomycin and fluoroquinolone are second-line TB drugs and are used only in special regimes of treatment, with the patient under close medical supervision. Testing cultures for susceptibility to first-line drugs was done at the Central Public Health Laboratories of *Mato Grosso do Sul* (*Laboratório Central de Saúde Pública de Mato Grosso do Sul*, Portuguese acronym LACEN-MS) only with clinical indication.

The indigenous territories included in this study (*Amambai, Guassuty, Jaguari, Kurussu Amba, Limao Verde, Taquaperi,* and *Caarapó*) are assisted by *Amambai* and *Caarapó* health units and are located on the border of Paraguay. Together, these Guarani-Kaiowá communities encompass approximately 18,000 people (Supplemental 1—Study characteristics).

### Tuberculosis isolate identification, regrowth and DNA extraction

Patients diagnosed with TB during the study period were identified in the records of the SESAI. *M. tuberculosis* isolates from these patients were identified in the MS Laboratory Environment Management System. All cultures still in storage at LACEN-MS were regrown in the Ogawa-Kudoh medium. DNA from the regrown cultures was extracted and purified at São Paulo’s Instituto Adolfo Lutz using the cetyltrimethylammonium bromide (CTAB)-lysozyme method as described by van Embden et al*.*^60^. Purified DNA concentrations were determined by Gene-Quant II (Pharmacia Biotech).

SESAI registered 277 incident cases during the study period. We set a cutoff for sample metadata completeness, which limited our set. Furthermore, not all samples arrived with sufficient quality for sequencing in Michigan, and WGS was ultimately performed on 68/277 samples.

### Whole-genome sequencing and barcoding

Sixty-eight samples were submitted for WGS on the MiSeq (Illumina) platform at Michigan State University. Samples were numbered S1–S68. Samples S02 and S36 failed quality checks and were excluded. The remaining 66 isolates was trimmed by Trimmomatic^61^ (trimmomatic PE, trimming TruSeq2 adapters, with parameters 2:30:10 LEADING:3 TRAILING:3 SLIDINGWINDOW:4:15 MINLEN:36) and reference-based assembly was performed using *M. tuberculosis* H37Rv in CLC Genomics Workbench v12.0. Representative global reference lineages included were *M. tuberculosis* 96121 (Manila-Lineage 1), HN878 (Lineage 2), HN-024 (Lineage 3), CDC1551 (Lineage 4), H37Rv (Lineage 4), Erdman (Lineage 4), and GM041182 (West African 2-Lineage 6). Multiple representatives of lineage 4 were included due to its historical and modern clinical importance. A whole genome multiple sequence alignment was constructed with default parameters in CLC (Supplemental 8—Alignment). To identify the sublineages of *M. tuberculosis* circulating in the community, we utilized specific SNPs for “barcoding” of TB, as reported by Coll et al*.*^16^. The whole-genome alignment of Brazilian isolates was searched manually for each of the 414 reported SNP barcodes. If all diagnostic SNPs for a particular sub-lineage were found in an isolate, it was considered part of that group. A summary is shown in Supplemental 2—SNP Barcoding for lineage identification.

### Core SNP extraction, phylogeny generation, and analysis

After initial analysis, we sought local references to better understand observed diversity. Unfortunately, assembled Brazilian whole genome sequences are vanishingly rare, and we reached out to Drs. Brynildsrud and Eldholm, who in 2018 were first and corresponding authors, respectively, on an expansive investigation into *Mycobacterium tuberculosis* lineage 4 isolates. Their dataset includes hundreds of paired-end FASTQ read sets for South American tuberculosis genomes. Reads for 9 Brazilian isolates unrelated to our current study were downloaded from NCBI (BioProject PRJEB27366) and processed utilizing the snippy pipeline^62^. Briefly, we included CLC-constructed contigs for all 66 of our isolates that passed QC, assembled whole genomes for global references, and raw paired-end FASTQ reads for 9 isolates (referred to in this manuscript as “local references”) from the Brynildsrud et al*.*, dataset (Supplemental 7—Isolates & References). These local references represent sublineages 4.3 (LAM, n = 7), 4.4 (S-type, n = 1), and 4.7 (Congo, n = 1)^63^. They were first processed by ABySS^64^ (abyss-pe, default parameters, k = 96) to construct contigs, and they, along with all contigs from our isolates and assembled genomes for global references, were batch-processed in snippy with the *M. tuberculosis* H37Rv Genbank (.gb) file as reference. The snippy output folders for each isolate and local references were processed by snippy-core using H37Rv.gb as reference. This output core alignment, comprised solely of variant sites and excluding complex changes like indels, was analyzed in 16 drug resistance-associated genes. To avoid the effects of uninformative, false positive hits in repetitive regions of the genome, snippy-core was run again with the mask parameter and the *M. tuberculosis* H37Rv-based .bed mask file included in the snippy package to filter out loci like PE/PPE family proteins. The unmasked and masked core SNP sets were compared, and ~ 3% of SNPs in both isolates and local references were considered uninformative by this approach. Masked core SNPs were passed into MEGA-X v10.1.8^65^ and MrBayes v3.2.7a^66^ separately. Maximum-likelihood trees were produced with 500 bootstrap replicates and the HKY model with other parameters default, and the tree with highest log-likelihood is shown (Fig. 1A). In MrBayes, the GTR model was used, with the parameter nst = mixed used to sample across the GTR model space. After a MCMC run length of 500,000, the standard deviation of split frequencies fell well below 0.01 as recommended, minimum ESS values were 300 or higher, above the 100 recommended, and PSRF values were ~ 1.000 (Supplemental 5—Bayesian Analysis). A quick comparison of ML phylogeny (HKY, default parameters, bs = 100) between masked and unmasked core SNPs showed greater bootstrap values for nodes derived from the masked set, suggesting removing uninformative hits yielded a more robust phylogeny (Supplemental 9—Masked vs Unmasked Phylogeny). As such, the masked SNP set was used for final analysis. Trees were visualized in FigTree and MEGA-X. Final labeling of trees (Fig. 1) was performed with Inkscape v.1.0^67^, but unmodified Newick tree files are also included for transparency (Supplemental 4 and 5).

### Ethical aspects

This study was performed in accordance with the Declaration of Helsinki and was approved by the Ethics Committee of the National School of Public Health (Report 354.060/2013) and the National Committee for Ethics in Research (Report 650.820/2014), which are both a part of the Brazilian Ministry of Health. Written informed consent was obtained from all study subjects.

## Supplementary Information

Supplementary Information 1.

Supplementary Information 2.

Supplementary Information 3.

Supplementary Information 4.

Supplementary Information 5.

Supplementary Information 6.

Supplementary Information 7.

Supplementary Information 8.

Supplementary Information 9.

## Data Availability

Raw FASTQ sequences for all 68 sequenced isolates (including the two that did not pass our QC) are freely available through NCBI (BioProjectID: PRJNA669332). This work would not have been possible without both global reference and Brazilian sequences also made available through NCBI. Accession IDs are available for all references in Supplemental 7 (Isolates & References).
